# Suicide assessment and feasible evidence-based treatments for adolescents living with HIV in Malawi: Protocol for a pilot randomized controlled trial

**DOI:** 10.1371/journal.pone.0330847

**Published:** 2025-09-03

**Authors:** Melissa A. Stockton, Katherine Waddell, Steven M. Mphonda, Nivedita L. Bhushan, James January, Charles Masulani, Gregory Brown, Michael M. Udedi, Brian W. Pence, Jonathan Chiwanda, Bradley N. Gaynes, Kazione Kulisewa

**Affiliations:** 1 Department of Epidemiology, Gillings School of Global Public Health, University of North Carolina at Chapel Hill, Chapel Hill, North Carolina, United States of America; 2 Psychiatry Department, Perelman School of Medicine, University of Pennsylvania, Philadelphia, Pennsylvania, United States of America; 3 University of North Carolina Project Malawi, Lilongwe, Malawi; 4 RTI International, Research Triangle Park, Durham, North Carolina, United States of America; 5 Psychiatry Department, Kamuzu University of Health Sciences, Blantyre, Malawi; 6 St. John of God Hospital, Mzuzu, Malawi; 7 NCDs and Mental Health Division, Ministry of Health, Lilongwe, Malawi; 8 Department of Psychiatry, University of North Carolina at Chapel Hill, Chapel Hill, North Carolina, United States of America; PLOS: Public Library of Science, UNITED KINGDOM OF GREAT BRITAIN AND NORTHERN IRELAND

## Abstract

**Background:**

This pilot trial will investigate the feasibility, acceptability, fidelity, and preliminary efficacy of a combined youth-friendly suicide prevention and problem-solving intervention in reducing suicidal ideation and behaviors in adolescents living with HIV (ALWH) in Malawi.

**Methods:**

ALWH (N = 60) aged 13–19 who reported suicidal ideation and behaviors will be recruited from 5 health clinics. Participants will be randomized 1:1 either to the Safety Planning and Friendship Bench (SP + FB) (n = 30) or to augmented usual care (n = 30) and followed for 6 months. SP + FB includes 6 individual counseling sessions and 6 bi-monthly peer support group sessions delivered by psychological counselors. Feasibility, acceptability, fidelity, and preliminary efficacy will be assessed at 6-weeks and 3- and 6-month study visits and compared across the 2 study arms.

**Discussion:**

The results of this study will provide insights into the feasibility, acceptability, fidelity, and preliminary efficacy of the combined SP + FB for reducing chronic and acute suicidality in ALWH compared to standard of care. This pilot study will lay the groundwork to test the SP + FB in a large-scale cluster randomized controlled trial to improve suicide prevention resources and interventions for ALWH in Malawi. The findings of the study will add to a critical body of research aiming to implement feasible, acceptable, and effective suicide prevention interventions as part of adolescent HIV care in low-resource settings.

**Trial registeration:**

ClinicalTrials.gov: NCT06770101

## Introduction

Suicide is the second leading cause of death worldwide for adolescents [1,2]. Nearly three-quarters of those suicides occur in LMICs [3] where up to 50–90% of suicides are associated with an underlying mental disorder, especially depression [4–6]. In Malawi, there are limited data on adolescent suicidal ideation and behaviors (SIBs) [7–9], but the estimated past-year prevalence of thinking about or attempting suicide is above 10% [10].

Adolescents living with HIV (ALWH) are at especially heightened risk of suicide, as ALWH grapple with living with a stigmatized chronic condition during a period of developmental transition, increasing their vulnerability to internalized stigma, self-esteem and mental health issues that may preclude suicidality [11–13]. While underlying depression is a common risk factor for SIBs among ALWH [11,14,15], other factors include struggling to accept their seropositive status, poor social support, decline in academic performance, poverty, familial bereavement, stigma, discrimination, and physical, sexual, and emotional abuse [16–23].

Suicide prevention efforts are lagging in Malawi. The significant toll of suicide in Malawi is garnering increased attention [24], and prompted the Malawi Ministry of Health to establish a Suicide Prevention Implementation Framework [25]. The Ministry’s Suicide Prevention Implementation Framework calls for the development of a national suicide prevention program aimed at improving access to adequate mental health care, strengthening legal policy to reduce discrimination, and supporting surveillance systems and suicide research [25]. Despite this policy-level support, Malawi still criminalizes suicide attempts, making them punishable with stiff custodial sentences [26,27]. Stigma around mental illness and suicide remains pervasive, resulting in suicidality concealment and barriers to care [28]. Further, psychiatric human resources in Malawi are limited – with four psychiatrists for 20 million individuals – which hampers prevention, identification, and management of SIBs [29]. As such, Malawi has relied on task-shifting and task-sharing to deliver mental health services – particularly for depression – in non-specialized settings such as HIV care [30,31]. Evidence suggests that task-shifting psychotherapy-based suicide prevention interventions, such as cognitive behavioral therapy (CBT) and problem-solving, are effective among people living with HIV in SSA [32–36]. However, comprehensive depression or SIBs screening and prevention services have yet to be integrated into adolescent HIV care, where standard of care for ALWH with SIBs (if identified) may include some supportive counseling or referral to tertiary level psychiatric facilities. This structural and socio-cultural context has created a landscape requiring innovations to address the burden of SIBs that will likely rely on leveraging existing mental health services to address the unique needs of ALWH [37].

The Friendship Bench (FB) is an evidence-based task-shifting counseling intervention that has demonstrated efficacy in improving mental health outcomes in low-resource settings, has been widely implemented in Malawi [30,31,38], and was recently adapted specifically for Malawian ALWH [36]. The FB is a lay health worker-delivered problem-solving therapy that teaches patients to effectively manage psychosocial stressors that may precipitate suicidality, by learning or reactivating problem-solving skills [32,39]. FB trials have demonstrated efficacy in addressing symptoms of common mood disorders in those with suicidality among adults [40]. Enhancing FB with specific, evidence-based suicide prevention activities may be a feasible and effective opportunity to meet the needs of ALWH with SIBs [36]. However, the FB has not yet been adapted to specifically address SIBs or meet the unique needs of ALWH with SIBs.

Accordingly, we augmented the existing adolescent-friendly FB model with Safety Planning (SP), a brief, evidence-based suicide prevention intervention based in CBT principles that targets acute suicidal behavior and can be integrated with existing healthcare services. The goal of SP is to reduce imminent risk of suicidal behavior through the collaborative development of a personalized list of coping strategies and resources for support during onset or worsening of suicidal crisis [41]. Further, SP can be delivered as a single-session intervention, with follow-up and is increasing being paired with problem-solving therapy to provide supportive follow-up. SP is ideal for the Malawian context, given it is one of the few suicide prevention interventions that has been used in an African setting [35], can be delivered by non-specialists, and can easily be incorporated into the existing FB problem-solving model to fully address chronic SIBs [42].

This paper aims to present the protocol for a randomized controlled trial designed to 1) investigate the feasibility, acceptability, and fidelity of the SP + FB intervention in addressing SIBs among ALWH; and 2) evaluate the preliminary efficacy of SP + FB compared to augmented usual care in reducing suicidal ideation and behaviors. If feasible, we will test the efficacy of the SP + FB intervention in a fully powered trial.

## Methods

### Overview of study design

**Fig 1:**
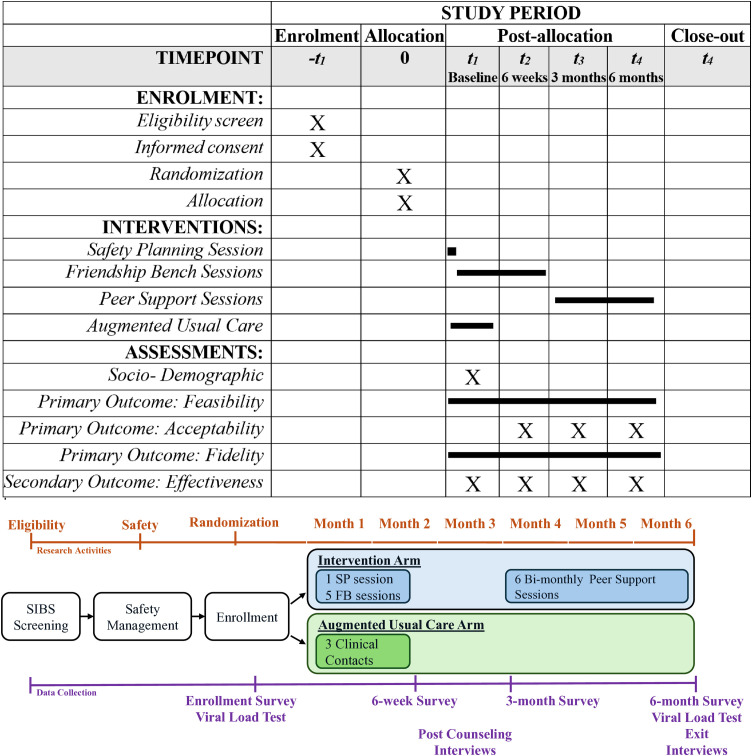
SPIRIT schedule. Study schedule of enrollment, interventions, and assessments.

We will conduct a two-arm individually randomized controlled pilot trial at five public HIV clinics in Malawi. Participants will be randomized 1:1 either to SP + FB (N = 30) or augmented usual care (N = 30) using a random sequence. This sequence will be designed using the rand() function in Microsoft 365 Excel version 2410). The study team will not release the sequence until after participants have completed enrollment to ensure allocation concealment. Recruitment began on 14 June 2025 and is planned through January 2026. Participants will be followed for 6 months after enrollment (Fig 1). We anticipate that data collection will be completed by August 2026. Results are expected to be available by December 2026.

### Study setting

The pilot trial will be conducted at five public health HIV clinics in Lilongwe, Malawi: Area 18, Area 25, Kawale, Likuni and Lighthouse Centers. These clinics were selected as provide ART services, are located in urban or peri-urban areas of Lilongwe District and hold quarterly adolescent ART clinic which serves >115 ALWH (ages 10–19) who are either initiating care, re-initiating care, or are established patients.

### Participants and sample size

Adolescents will be recruited from the five study clinics and eligible for the study if they are: (1) age 13−19; (2) diagnosed with HIV (vertically or horizontally acquired); (3) report SIBs on the Ask Suicide Questions (ASQ) [43] and/or question 9 of Patient Health Questionnaire-9-Adolescent (PHQ-9-A) [44]; (4) living in the clinic’s catchment area with intention to remain for >1 year; and (5) willing to provide consent (age 18+ or 16−17 years old and married and thereby considered emancipated minors per Malawi law) or assent with parental consent (age 13−17). Any individual who reports SIBs (regardless of eligibility or trial enrollment) will be offered suicide safety management care. We expect to enroll 60 participants for this pilot study (30 per arm). As a feasibility pilot, the sample of 60 ALWH (30 per arm) is powered to be sufficient to estimate quantitative measures of feasibility and acceptability with reasonable precision (e.g., standard errors of.06−.09 for proportions).

### Intervention description

The SP + FB protocol integrates SP within the existing adolescent-friendly FB model, tailored for ALWH in the Malawian context [36,45]. Prior to implementation, the protocol was adapted based on qualitative feedback from the formative phase of the study in which ALWH, their caregivers, healthcare staff and leadership, and other key advocates to ensure that the SP meets the specific suicide prevention needs of ALWH in the Malawian cultural and legal context. The protocol will include six sessions (1 SP session and 5 FB sessions) delivered by trained psychosocial counselors followed by six peer-support sessions (PS) delivered by trained peer supporters living with HIV (**Box 1**). SP + FB counselors will be psychosocial counselors (mixed genders, aged 20–35) who are motivated to work with young people. Counselors will attend a two-week training on SP and FB problem-solving therapy, and deliver the integrated SP + FB program. Additionally, the SP + FB counselors will be trained to deliver non-judgmental therapy that respects the privacy and autonomy of ALWH and enables ALWH to make free and informed choices that are relevant to their individual needs. Training will also include discussion of adolescent development and the unique experiences of ALWH related to stigma, disclosure, social relationship issues, and health care access. Therapy sessions will take place in a youth-oriented but discrete private clinic room within the HIV clinic and be available on weekends to ensure accessibility.

#### Box 1

Intervention schedule.

Session 1 (Week 1; 45–60 min)
  - SIBs & Suicide Risk Assessment
  - Psychoeducation & SP + FB Introduction
  - SP activities & Strategy Creation
Sessions 2 (Week 2; 40–45 min)
  - SIBs & Suicide Risk Assessment
  - SP Check-in & Revision
  - FB Problem-Solving Initiation
Sessions 3–6 (Weeks 3–6; 15–20 min)
  - SIBs & Suicide Risk Assessment
  - SP Check-in & Revision
  - FB Problem-Solving Continuation
Sessions 7–12 (Months 3–6, 90 min)
  - Peer support groups sessions
Optional Support
  - Telephone/text message check-ins

SP will be initiated during the first session and will consist of an introduction to the program, a suicide risk assessment of SIBs including a narrative assessment of a suicidal crisis, and the collaborative development of a personalized list of coping strategies and resources. Session 1 is expected to last 45–60 minutes. SIBs and suicide risk will continue to be assessed at follow-up sessions, with optional telephone and/or text message check-ins.

FB problem-solving counseling will begin during the second session. The FB is a highly structured therapeutic technique delivered by lay counselors who guide clients through a series of steps. First, the counselor works with the client to identify prior maladaptive coping strategies. The counselor then works with the client to explore and recognize various psychosocial problems that may be adversely impacting the client’s mental health and suicide risk, ultimately arriving at a ‘priority problem’ that the client believes should be the focus of the problem solving. The counselor subsequently encourages the client to brainstorm for range of solutions to the identified problem, discussing the feasibility and likelihood of each potential solution, and aiding the client to eliminate impracticable suggestions to arrive at an attainable solution. The client and counselor collaboratively develop a specific, measurable, achievable, relevant and time bound (SMART) plan to achieve the desired solution. Subsequent sessions are similarly iterative, with the addition of an evaluation step where the prior session’s SMART plan is appraised in light of whether the client succeeded in resolving their priority problem or whether there were missteps or barriers the client hadn’t factored in. Through these 30–45-minute sessions, the counselor aids the client in addressing prior dysfunctional coping strategies through behavioral activation and developing problem solving skills. The last two sessions will last 15–20 minutes and serve as a review and check-in on the SP and discussions that occurred in previous FB sessions. No specific retention support will be provided, but participants may identify barriers to engagement in HIV care to address during their counseling session. After the 4 sessions of individual therapy, the counselor can refer participants with worsening or unresolved SIBs to a psychiatric specialist (study psychiatrist or psychologist) for reassessment and safety management. Case management may include additional counseling, pharmacotherapy, and/or referral for in-patient psychiatric care at the discretion of the managing clinician.

Additionally, ALWH in the intervention arm will receive peer support (PS) via in-person 6 group meetings to facilitate engagement in care for ALWH and support SIBS reduction, as is now standard with the existing FB package adapted for ALWH in Malawi [36]. Peer supporters will be mature young adults (aged 18–21) who have completed secondary school, are openly living with HIV, and motivated to work with adolescents. The peer supporters must be willing to maintain participant and colleague confidentiality and sign a confidentiality agreement. Peer supporters will be trained to deliver youth-friendly group sessions related to HIV care. The content of the 6 peer support sessions includes: Mental Health and HIV, Status Communication, Adherence and Viral Load Testing, Secondary Prevention (Sex and Relationships), Stigma, and Planning for the Future. The 6 sessions will be delivered following completion of the SP + FB sessions and will be held bi-monthly over 3 months and last 90 minutes each. The group sessions will be in a private space in the clinic in the participant’s local language (Chichewa).

### Augmented usual care

Augmented usual care will include all the following elements of the standard of care for suicidality in public health facilities in Malawi: assessment, basic supportive counseling by the primary provider or nurse, antidepressant medication management by the primary provider if deemed necessary, referral to the clinic’s psychiatric nurse, or, for acute cases or crises, referral to the psychiatric units at tertiary care hospitals (Kamuzu Central Hospital in Lilongwe) at enrollment. Augmented usual care will be provided to the intervention arm. Nurses and clinicians at the study sites have been specifically trained to use the Tool for Assessment of Suicide Risk for Adolescents (TASR-A) to assess ALWH considered at elevated risk for suicide for all study arms. For this study, usual care will be augmented by a trained study nurse who will provide mental health evaluation, brief supportive counseling, information, education, and support on SIBs, and (if indicated) facilitation of referral to the clinic’s psychiatric nurse or to Kamuzu Central Hospital under the supervision of the study psychiatrist. The study nurse will have follow-up contacts (in-person or by phone) with the participant to assess whether the participant has followed up on recommended referrals or treatment plans and to assess whether any further outreach is needed.

### Primary and secondary outcomes

The primary outcomes of this pilot study are to evaluate the feasibility, acceptability, and fidelity of the SP + FB intervention to address SIBs among ALWH. Indicators of preliminary efficacy of the intervention for improving mental health will be reported as secondary outcomes. Preliminary efficacy outcomes will be assessed at 6-weeks and 3- and 6- month study visits and compared across the two study arms. Additionally, we will conduct brief exit interviews with the SP + FB counselors (n = 4), the peer supporters (n = 4), and a sub-set of enrolled ALWH (n = 10). For the ALWH, exit interviews will occur after the final SP + FB session (n = 5; approximately 6–9 weeks after beginning the intervention) and after the completion of peer support sessions (n = 5; approximately 6–7 months after beginning the intervention). Exit interviews with the interventionists will occur after the end of the study activities at each site.

*Feasibility* will be defined as the ability to successfully enroll and retain ALWH with SIBs in the study and the number of sessions (SP + FB and peer-support) attended during the study period.

*Fidelity* will be defined as adherence to the SP + FB protocol. Fidelity to content for SP + FB sessions will be reviewed by a member of the study team using audio recordings of the sessions. The SP session fidelity checklist was adapted from Brown & Stanley’s SP Intervention fidelity measure for adolescents which consists of 14 questions rating the completion of pre-SP activities and the quality of general SP intervention skills and the conduct of each step of the Safety Plan [46]. The FB sessions checklist consists of 11 items corresponding to 11 core session components rating from “1 – Component not done” to “4 – Component exceeds expectations” for each component. Covering at least 80% of checklist items will be considered fidelity to the intervention protocol [36,47].

*Acceptability* will be defined as the ability of counselors to deliver and patients to participate in a useful intervention that is appropriate regarding resources and culture. Acceptability will be assessed quantitively using the Adapted Client Satisfaction Questionnaire-8 [48,49] and qualitatively in exit interviews which have both been used in similar pilot trials for adolescents in Malawi. The Adapted Client Satisfaction Questionnaire-8 consists of 8 items adapted for youth measuring the level of satisfaction with an intervention. Participants are asked to rank their satisfaction from 1–4 with the intervention overall, the quality of care they received, how the intervention met their expectations and needs, and how the intervention helped them, and then were also asked about whether they would be likely to use the intervention again and recommend their friends. The scores to each question are summed with total scores ranging from 8–32 and higher scores indicating greater satisfaction. Exit interviews will include both closed and open-ended questions exploring participants’ satisfaction level, how easy the intervention was to participate in or deliver, the perceived usefulness of the intervention, suggestions for improvement, and will explore contextual factors that impeded or facilitated implementation.

*Preliminary Efficacy* will be assessed through changes from enrollment to 6 weeks, 3 months and 6 months in the prevalence of: 1) past two-week suicidal ideation (any yes response to PHQ9-A Q9, ASQ1, ASQ2, or ASQ3); 2) current suicidal intent (yes response to ASQ5); 3) suicide risk (acute or moderate to high vs low or no) as measured through the locally developed Suicide Risk Assessment Protocol [29]. Both the PHQ-9A and the ASQ have been used in this setting for ALWH. The PHQ-9A Q9 asks participants for the frequency of which they experienced suicidal ideation in a two-week period from “Not at all” to “Nearly Everyday”. The ASQ Q1, Q2, and Q3 ask participants “Yes” or “No” questions about their suicidal ideation within the past few weeks, whereas Q5 asks participants about their current suicidal ideation.

### Data collection

For quantitative data for primary implementation outcomes, study staff will collect data related to enrollment, retention, and session attendance. Additionally, fidelity will be assessed by the research team using a checklist of intervention characteristics when reviewing audio-recorded sessions. Quantitative data will be collected and managed using Research Electronic Data Capture (REDCap).

For the qualitative data for primary outcomes, exit interviews will be digitally recorded, transcribed in Chichewa, and then translated into English by study staff according to a transcription protocol. All transcripts will then be reviewed by the interviewer for transcription and translation accuracy.

For preliminary efficacy outcomes, study staff will collect SIBs data from survey participants at enrollment, 6-weeks, 3-months and 6-months using REDCap. The survey questionnaire will include information related to socio-demographic characteristics, SIBs, mental health, social support, HIV care and stigma (Table 1). Viral load testing will be conducted at enrollment and 6-months. All participants will receive 10 USD for each study visit and intervention arm participants will receive 4 USD as transport reimbursement for attending counseling and peer support sessions.

**Table 1:** Assessment domains, constructs, and measures.

Domain	Construct	Measures	Assessment Point	Data Type
**Primary Implementation Outcomes**				
	Feasibility	# enrolled; # planned vs. # actual enrollment; reasons for non-enrollment; % retained in each arm; # of sessions attended	Ongoing	Clinic
		In-depth interviews	Exit Interview	Qual.
	Fidelity	Checklist for content covered during sessions	Ongoing	Clinic
	Acceptability	Adapted Client Satisfaction Questionnaire-8 [49]	FU1–3	Survey
		In-depth interviews	Exit Interview	Qual.
**Secondary Preliminary Efficacy**				
	SIBs	PHQ-9-A Question 9 and ASQ Tool [43,50]	Ongoing; BL, FU1–3	Clinic; Survey
	Suicide Risk	Suicide Risk Assessment Protocol [29]		
**Covariates & Mediators**				
	Demographics	Age, sex, resources, education, religion, occupation, health status, awareness of HIV status at ART initiation, home distance	BL	Survey
	Disclosure	HIV and Suicidality Disclosure to Family and/or Friends	BL	Survey
	MH Care Engagement	SP + FB appointment dates, other MH treatment	Ongoing	Clinic
	HIV Care Engagement	ART appointment dates, ART pill count, VL draws	Ongoing	Clinic
	HIV Stigma	Internalized AIDS-Related Stigma Scale [51]	BL, FU1–3	Survey
	Suicide Stigma	Personalized Suicide Stigma Questionnaire [52]	FU1–3	Survey
	Suicide Coping	Suicide Related Coping Scale [53]	BL, FU1–3	Survey
	Isolation	Adapted Interpersonal Needs Questionnaire items [54]	BL, FU1–3	Survey
	Burdensomeness	Adapted-Suicide Cognitions Subscale items [55]	BL, FU1–3	Survey
	Hopelessness	Adapted-Beck Hopelessness Scale items [56,57]	BL, FU1–3	Survey
	Acquired Capability	Adapted-Acquired Capability for Suicide Scale items [54]	BL, FU1–3	Survey
	Sleep Quality	Pittsburgh Sleep Quality Index [58]	BL, FU1–3	Survey
	Depressive Symptoms	PHQ-9-A [44]	Ongoing	Clinic
	Trauma	PC-PTSD-5 [59]	BL, FU1–3	Survey
	Alcohol/Marijuana	Single-question alcohol screening test [60]; single-item screen-cannabis [61].	BL, FU1–3	Survey
	Social Support	Multi-dimensional Scale of Perceived Social Support [62,63]	BL, FU1–3	Survey
	Resilience	PLHIV Resilience Scale [64]	BL, FU1–3	Survey
**NIH Common Data Elements**				
	Anxiety, Depression, Functioning	Revised Children’s Anxiety [65] and Depression Scale-25 (RCADS-25) Youth Assessment [66]	BL, FU1–3	Survey
	DSM-5 Cross-cutting	DSM-5 Cross-cutting assessment Youth Self Report [67,68]	BL, FU1–3	Survey

BL = Baseline; FU = Follow-up; FU1 = 6 weeks; FU2 = 3 months; FU3 = 6months; Ongoing = abstracted from clinical records from counseling sessions or HIV care appointments

### Data analysis

Baseline characteristics of participants will be compared between the arms using t-tests for continuous variables and chi-square tests for categorical variables to assess balance across study arms.

Quantitative measures of the primary outcomes, feasibility, acceptability, and fidelity will be summarized using means and standard deviations or proportions and compared across arms using statistical models for continuous or binary outcomes, as appropriate. Specifically, we will compare the number of people screened and then enrolled and the proportion retained in each arm through 6 months (feasibility); the proportion of participants who found the intervention easy to administer and helpful Adapted Client Satisfaction Questionnaire-8 (acceptability) [32], and the proportion of interventionists covering at least 80% of checklist items during random direct monitoring sessions (fidelity).

Qualitative measures of acceptability (e.g., open-ended questions on the exit interview) will be analyzed using textual data analysis: reading for content; coding; data display; data reduction; and interpretation [69].

The secondary outcome of preliminary efficacy will be analyzed at 6-weeks and 3- and 6-months and compared between study arms. In addition, statistical models examining differences in prevalence of SIBs and suicide risk will account for baseline values by using generalized estimating equations with an exchangeable correlation matrix. Importantly for the design of the subsequent RCT, this pilot will yield estimates of the within-clinic intra-cluster correlation and the standard deviation for the efficacy outcomes.

### Ethics and confidentiality

The Institutional Review Boards of the University of North Carolina at Chapel Hill and the Malawi National Health Sciences Research Committee (NHSRC) approved this study. All eligible and interested participants aged 18 and above or legally emancipated 16–17-year-old married minors, will provide written informed consent. All eligible and interested participants aged 13–17 will provide written assent with written parental consent. All participants (and guardians if present) will receive travel reimbursement equivalent to 10 USD for participating in a study visit and 4 USD for attending counseling and peer support sessions.

## Discussion

Malawian ALWH face unique challenges due to living with a chronic, stigmatized disease in a low-resource setting that increased their risk of SIBs as they navigate the transition from childhood to adulthood. Though suicide exacts a heavy mortality toll particularly among adolescents in LMICs like Malawi, limited psychiatric specialists and the continued criminalization of suicide have led to a lack of concerted suicide prevention efforts in Malawi [24]. Additionally, researchers have found that passive suicidal ideation can manifest in refusal to adhere to HIV care in adolescents [70,71]. Addressing SIBs is particularly important due to the growing ALWH population and the increasing rate of mortality among ALWH in in sub-Saharan Africa [72]. Suicide prevention interventions similar to SP have demonstrated efficacy and feasibility in the African setting for reducing acute suicidal crises [35]. Additionally, SP can be delivered by non-specialists and can easily be incorporated into existing services to fully address chronic SIBs, making it ideal for delivery in Malawi [42]. The Friendship Bench intervention has also been successfully adapted for meeting the mental healthcare needs of ALWH in Malawi [33,36]. In prior studies conducted by our team, we found that experience, respectful young counselors, time flexibility, and confidentiality in location and counselor are crucial for a youth-friendly adapted model of FB [33]. Taken together, such evidence suggests that SP + FB may be both feasible and effective in the Malawian setting for ALWH. Accordingly, the current pilot randomized controlled trial will provide critical data into feasibility and preliminarily efficacy of SP + FB for ALWH in Malawi. Namely, this pilot will contribute to the body of evidence for the potential feasibility, fidelity, acceptability, and preliminary efficacy in addressing chronic and acute SIBs of the SP + FB intervention for ALWH in Malawi. Finaly, this pilot will provide insight into the suicide prevention services for ALWH in Malawi and will support efforts to enhance clinical capacity to identify and manage suicide risk.

The study may be subject to several potential limitations. Since the SP + FB model is new to Malawi, this pilot may face challenges controlling consistency of the intervention delivery across the five clinics. To combat this challenge, we are using a Training-of-Trainers (TOT) model to develop local Malawian expertise in SP to both train and supervised the SP + FB counselors. Additionally, the SP and FB sessions will be recorded, and supervisors will use the recordings to assess fidelity and provide ongoing feedback and capacity-building support. We expect the intervention consistency across clinics to be met through intensive training and supervision. Additionally, suicide criminalization will continue to hamper concerted suicide prevention efforts in Malawi. This pilot trial will yield data on the feasibility of offering age-appropriate evidence-based suicide prevention services to ALWH in a setting lacking supportive legislation. Such data should be utilized in the larger decriminalization conversation, and leveraged to bring policy makers, clinicians, public health researchers, legislators and other key stakeholders together to tackle the complex issue of suicide criminalization. Finally, the generalizability of the findings of this pilot RCT may also be limited by the lack of diversity of the sample given that all ALWH will be recruited from urban or peri-urban areas within Lilongwe District.

## Conclusion

ALWH in LMICs like Malawi are at elevated risk of suicide, necessitating resource-appropriate interventions to address both chronic and acute SIBS. This pilot trial will provide insights into the feasibility of a youth-friendly SP + FB intervention and its ability to address the unique needs of ALWH with SIBs in Malawi. The study will generate fundamental new knowledge into the delivery of an evidence-based suicide prevention intervention, laying the groundwork for a future large-scale cluster randomized controlled trial to reduce suicide risk as well as to improve mental health and HIV care engagement among ALWH.

## Supporting information

S1 Table SPIRIT 2013 Checklist: Recommended items to address in a clinical trial protocol and related documents*. (DOCX)

S1 File Study protocol (SAFETY Planning). (DOCX)
