# Supplementary material for: Suicide assessment and feasible evidence-based treatments for adolescents living with HIV in Malawi: Protocol for a pilot randomized controlled trial
Source: PLoS One. 2025 Sep 3;20(9):e0330847. doi: 10.1371/journal.pone.0330847 (PMC12407430; doi:10.1371/journal.pone.0330847)
Supplement: S1 File — (DOCX) [file pone.0330847.s002.docx]

**Suicide Assessment and Feasible Evidence-based Treatments for Youth Living with HIV in Lilongwe: SAFETY Planning Pilot Trial**

**Sponsored by:**

**National Institute of Mental Health (NIMH) at the National Institutes of Health**

**Version 4.0**

**04 April 2025**

# ABSTRACT

Suicide exacts a heavy mortality toll worldwide, especially among adolescents in low- and middle-income countries (LMICs). In Malawi, there are limited data on suicidal behavior, though the estimated past-year prevalence of thinking about or attempting suicide among adolescents is above 10%. While the key factors driving suicide among adolescents remain unknown, adolescents living with HIV (ALWH), particularly those with co-morbid depression, are a vulnerable group at heightened risk of suicidal ideation and behaviors (SIBs). Resource-appropriate interventions that address SIBs among ALWH are urgently needed.

Safety Planning (SP) is a brief, evidence-based suicide prevention intervention that targets acute suicidal behavior and can be easily integrated with existing services, making it a superb candidate for such an adaptation to address this gap in care. SP is ideal for Malawi, given it is one of the few suicide prevention interventions that has been used in an African setting, can be delivered by non-specialists, and can easily be incorporated into existing services to fully address chronic SIBs.

The Friendship Bench (FB) is an evidence-based depression counseling intervention delivered by trained, supervised lay health workers. The FB approach is based on problem-solving therapy principles, making it an ideal candidate for enhancement with additional, evidence-based practices to more holistically address chronic and acute SIBs.

This project aims to evaluate the feasibility, acceptability, fidelity, and preliminary effectiveness of the enhanced FB+SP model through a pilot randomized controlled trial.

TABLE OF CONTENTS

[ABSTRACT 2](#_Toc172903136)

[1.0 BACKGROUND, PROBLEM STATEMENT, AND JUSTIFICATION 6](#_Toc172903137)

[1.1 Background and Literature Review 6](#_Toc172903138)

[1.2 Problem Statement 6](#_Toc172903139)

[2.0 MAIN AND SPECIFIC OBJECTIVES 7](#_Toc172903140)

[2.1 Main Objective: 7](#_Toc172903141)

[2.2 Specific Objectives: 7](#_Toc172903142)

[3.0 HYPOTHESES 7](#_Toc172903143)

[4.0 LITERATURE REVIEW 7](#_Toc172903144)

[5.0 METHODOLOGY 9](#_Toc172903145)

[5.1 4.1 Design of Study 9](#_Toc172903146)

[5.2 Place of study 10](#_Toc172903147)

[5.3 Target Population 10](#_Toc172903148)

[5.4 4.4 Sampling Techniques and Tools 10](#_Toc172903149)

[5.5 4.5 Sample Size Determination 10](#_Toc172903150)

[5.6 4.6 Data Collection Techniques and Tool 11](#_Toc172903151)

[5.7 4.7 Data Analysis 12](#_Toc172903152)

[5.8 Dissemination of Results 12](#_Toc172903153)

[6.0 ETHICAL CONCERNS 12](#_Toc172903154)

[6.1 Human Subjects 12](#_Toc172903155)

[6.2 Institutional Review Board 13](#_Toc172903156)

[6.3 Confidentiality, Risks, and Risk Minimization 13](#_Toc172903157)

[6.4 Benefits to Participants 15](#_Toc172903158)

[6.5 Costs and Compensation 15](#_Toc172903159)

[6.6 Informed Consent 15](#_Toc172903160)

[6.7 5.2 Adverse Event Reporting 16](#_Toc172903161)

[6.8 Study Discontinuation 16](#_Toc172903162)

[6.9 Amendments to Protocol 16](#_Toc172903163)

[6.10 Record Retention 17](#_Toc172903164)

[7.0 PERSONNEL ROLES AND INSTITUTIONS 17](#_Toc172903165)

[7.0 WORKPLAN 18](#_Toc172903166)

[8.0 BUDGET 18](#_Toc172903167)

[8.1 Budget Justification (USD)  19](#_Toc172903168)

[9.0 REFERENCES 21](#_Toc172903169)

HISTORY OF PROTOCOL CHANGES

Version 1.0, 4 April 2024

- None to date

Version 2.0 7 November 2024

- Fixed spelling and grammatical mistakes
- Section 2.0
  - Retitled the patient outcomes section to “"Preliminary Effectiveness” and explained how patient outcomes relateto preliminary effectiveness
  - The main objective was clarified and revised
  - The second specific objective was revised to be clearer and reduce confusion regarding what elements are being compared
- Section 3.0
  - A hypothesis was added
- Section 5.2
  - Updated to reflect that the study will occur across four clinic sites rather than three
- Section 5.5
  - Information on qualitative interviews was added
- Section 5.6
  - Patient Outcomes retitle “Preliminary Effectiveness”

Version 3.0, 13 January 2025

- - Section 6.7
    - Removed Data Safety Monitoring Board (DSMB) from protocol
  - Section 5.6
    - Added detail on VL draws as part of preliminary effectiveness
    - Information on VL data collection was added to Table 1.
  - Section 8.0
    - Add cost of VL testing
  - Glossary of Terms/Acronyms
    - “VL- Viral Load” was added

Version 4.0, 04 April 2025

- - - Section 1.2
      - Changed “four facilities” to “five” to reflect the additional study site
    - Section 5.1
      - Changed “four public health clinics” to “five” to reflect the additional study site
    - Section 5.2
      - Changed “four public health clinics” to “five” to reflect the additional study site
      - Added “Likuni” health center to the list of study sites
    - Section 5.3
      - Changed “four study clinics” to “five” to reflect the additional study site
      - Changed N=20 to N=12 to reflect the addition of the study site
    - Section 5.4
      - Changed “four HIV clinics” to “five” to reflect the additional study site

GLOSSARY OF TERMS/ACRONYMS

ALWH- Adolescents living with HIV

ASQ- Ask Suicide-Screening Questions

CAB- Community Advisory Board

DSMB- Data Safety Monitoring Board

FB- Friendship Bench

IDI- In-depth interview

LMIC- Low- and middle-income countries

NHSRC- National Health Sciences Research Committee

PHQ-9- Patient Health Questionnaire-9

PHQ-9/A- Patient Health Questionnaire-9/Adolescents

PO- Program officer

SAE- Serious adverse events

SIB- Suicidal ideation and behaviors

SP- Safety Planning

SRAP- Suicide Risk Assessment Protocol

VL – Viral Load

# BACKGROUND, PROBLEM STATEMENT, AND JUSTIFICATION

## Background and Literature Review

Suicide exacts a heavy mortality toll worldwide, especially among adolescents in low- and middle-income countries (LMICs).^1^ Suicide is the second leading cause of death worldwide for adolescents.^2,3^ Nearly three-quarters of those suicides occur in LMICs,^4^ where up to 50- 90% of suicides are associated with an underlying mental disorder, especially depression.^5-7^ In Malawi, there are limited data on adolescent suicidal ideation and behaviors (SIBs) ^8-10^ but the estimated past-year prevalence of thinking about or attempting suicide is above 10%.^11^ Adolescents living with HIV (ALWH), particularly those with co-morbid depression, are at especially heightened risk of suicide and in urgent need of intervention.^12^

Despite the significant toll of suicide in LMICs, prevention efforts are lagging in Malawi. While the Malawi Ministry of Health has called for the development of a national suicide prevention program,^13^ Malawi still criminalizes suicide attempts, making them punishable with stiff custodial sentences.^14,15^ Stigma around mental illness and suicide remains pervasive, resulting in suicidality concealment and barriers to care.^16^ Psychiatric human resources in the country are limited – with four psychiatrists for 20 million individuals – which hampers prevention, identification, and management of SIBs.^17^ This structural and socio-cultural context has created a landscape requiring innovations to address the burden of suicidality.

## Problem Statement

The Friendship Bench (FB) is an evidence-based intervention that is highly effective for depression, which is a major determinant of suicidality. FB is a lay health worker-delivered problem-solving therapy that teaches patients to effectively manage psychosocial stressors that may precipitate suicidality, by learning or reactivating problem-solving skills.^18,19^ FB trials have demonstrated efficacy in addressing symptoms of common mood disorders in those with suicidality.^20^ The FB model was recently adapted for ALWH Malawi (R34MH130232; MPI Bhushan/Gaynes). Enhancing FB with specific, evidence-based suicide prevention activities may be a feasible and effective opportunity to meet the needs of ALWH with SIBs.

Safety Planning (SP) can enhance the FB’s suicide prevention capability. SP is a brief, evidence-based suicide prevention intervention that targets acute suicidal behavior and can integrate with existing services. SP derives from cognitive-behavioral therapy for suicide prevention,^21^ and aims to reduce imminent risk of SIBs by co-creating personalized coping strategies to be utilized in a suicidal crisis to avert suicidal thoughts and manage suicidal urges.^22^ As few evidence-based suicide prevention interventions have been implemented in Africa,^23^ SP is ideal for Malawi, given it has been delivered by non-specialists and can easily be incorporated into existing services to fully address chronic SIBs.

This project aims to evaluate the feasibility, acceptability, fidelity, and preliminary effectiveness of the enhanced FB+SP intervention model. We will enroll 60 depressed ALWH who report suicidality from five facilities in Lilongwe, Malawi, and randomize them 1:1 to the enhanced FB+SP model or augmented usual care. This pilot trial is a step toward our long-term goal of generating and implementing an evidence-based model to prevent suicide in Malawi amongst ALWH by enhancing the capacity of the health system to identify suicidality and provide evidence-based care. Information gathered in this proposal will be used to develop a subsequent RCT.

# MAIN AND SPECIFIC OBJECTIVES

## Main Objective:

- The overall aim of this study is to determine the feasibility, fidelity, acceptability, and preliminary effectiveness of the FB+SP intervention in reducing SIBs and improving HIV engagement amongst adolescents living with HIV (ALWH) when compared to augmented usual care.

## Specific Objectives:

- To determine feasibility, fidelity, and acceptability of the FB+SP protocol
- To determine the preliminary effectiveness of the FB+SP protocol in reducing SIBs and in improving engagement in HIV care among ALWH compared to augmented usual care

# HYPOTHESES

We hypothesize:

- The FB+SP intervention will be feasible, delivered with fidelity and acceptable.
- ALWH in the FB+SP intervention arm will demonstrate a greater reduction in SIBs and improvements in HIV care engagement compared to ALWH in the augmented usual care arm.

# LITERATURE REVIEW

Growing literature, mostly involving adults, suggests that non-psychiatric specialists in resource-limited settings can deliver evidence-based mental healthcare, including services for SIBs.^23-25^ These task shifting approaches have been used in SSA and in Malawi specifically, which has few psychiatric specialists and has relied on task-shifting and task-sharing to expand access to depression care.^26,27^ Such task-shifting approaches to depression care move beyond a purely biomedical approach to mental health, often incorporating sociocultural frameworks and counseling therapy.^28-30^ However, existing depression interventions incorporating these approaches have been largely developed for adult populations and few have specifically targeted SIBs. ^23,31^ Adolescence is a unique period of profound physical, cognitive, and social change where individuals begin to make health-related decisions independently but are still largely influenced by their family members, peers, and intimate partners. ^31^Navigating these developmental changes is further complicated for ALWH who face issues surrounding inconsistent support from their caregivers, negotiating safe sex, status disclosure to their social ties, stigma related to HIV, and barriers in accessing health services.^32-34,35-37^ ^38^ Considering the complex needs of ALWH, there has been a global push for both physical and mental health interventions which are accessible, confidential, and developmentally appropriate, and which provide a safe space where adolescents can share sensitive concerns without judgement.^39^ While most existing youth-friendly intervention models have largely focused on sexual and reproductive health, ensuring mental health services – particularly those for depression and SIBs – are culturally-appropriate and youth-friendly is equally critical.^40,41^ Taken together, addressing SIBs in low-resources settings will require leveraging existing mental health services and rigorously adapting interventions to address the unique needs of adolescents.

Despite the significant toll of suicide, the expansion of mental health services, and Ministry of Health policy prioritization, limited psychiatric specialists and the continued criminalization of suicide have led to a lack of concerted suicide prevention efforts in Malawi. The rising incidence of suicide in Malawi has brought increased attention to the matter.^42^ In light of this reality, the Malawi Ministry of Health has recently finalized a national Suicide Prevention Implementation Framework.^13^ This framework formally prioritizes the establishment of a national suicide prevention program; efforts to strengthen the legal environment to support suicide prevention; improved access to comprehensive, integrated, and quality services for suicide interventions at the primary care level; increased awareness of suicide and suicide prevention; reduction of stigma and discrimination; and strengthen surveillance systems and suicide research. However, Malawi still criminalizes suicide in Section 229 of the Penal Code, rendering suicide attempts punishable with stiff custodial sentences.^14,43^ Additionally, Malawi has limited mental health infrastructure and few psychiatric specialists;^26^ the four mental health facilities in the country are all located in urban centers, and mental health care is still largely a specialized service, primarily offered from these facilities. As such, suicide prevention efforts, care seeking, and available services in Malawi are lagging considerably. Investment in evidence-based practices for non-specialists are urgently needed to address these gaps.

Safety Planning (SP) is a brief, evidence-based suicide prevention intervention that targets acute suicidal behavior and can be easily integrated with existing services, making it a superb candidate for such an adaptation to address this gap in care. SP is derived from cognitive-behavioral therapy for suicide prevention, with the ultimate goal of reducing imminent risk of suicidal behavior through the co-creation of a personalized list of coping strategies for support during onset or worsening of suicide-related distress. In the event of a suicidal crisis, an individual may use these strategies to support themselves, avert their suicidal thoughts and manage their suicidal urges.^22^ SP includes six primary elements: (1) identifying warning signs of a suicide crisis; (2) internal coping strategies; (3) social supports that can distract from the current crisis; (4) contact information for these social supports; (5) contact information for health care services; and (6) reducing access to lethal means.^22^ SP is ideal for Malawi, given it is one of the few suicide prevention interventions that has been used in an African setting,^23^ can be delivered by non-specialists,^25^ and can easily be incorporated into existing services to fully address chronic SIBs.

The Friendship Bench (FB) is an evidence-based task-shifting counseling intervention that has demonstrated efficacy in improving mental health outcomes in low-resource settings and was recently adapted for ALWH in Malawi. The FB was developed over 20 years of community research to provide lay health workers with the tools, training, and supervision to effectively address depression and other common mental health disorders in Zimbabwe.^18,44^ The FB was originally intended for use in primary care settings for the general adult population, although youth-friendly adaptations appear acceptable for meeting the mental healthcare needs of young people.^45,46^ In Malawi, our team has led efforts to pilot test expansions of the FB for adults and adolescents with HIV, patients with chronic disease, and perinatal women.^26,27,47-49^ These expansions have incorporated a locally adapted version of the Patient Health Questionaire-9 (PHQ-9)^50^ augmented with a Suicide Risk Assessment Protocol that is used to assess suicide risk by differentiating active from passive SIBs and referring patients for intensive psychiatric treatment as necessary.^17^ However, the FB has not been adapted to specifically address SIBs or meet the unique needs of ALWH with SIBs. Further, in Malawi, skilled counselors are not widely available, are often middle aged, and not trained to specifically identify suicide risk. Thus, we propose to integrate evidence-based suicide prevention practices into the FB intervention using young counselors also trained to deliver SP.

The FB approach is based on problem-solving therapy principles, making it an ideal candidate for enhancement with additional, evidence-based practices to more holistically address chronic and acute SIBs. The FB comprises 6 individual sessions delivered by trained, supervised lay counselors attached to a primary care clinic. The FB uses problem-solving therapy to improve mental health outcomes by having counselors work with participants to identify a problem and possible solutions.^51,52^ The FB was tested in a cluster-randomized controlled trial in 24 primary care clinics in Zimbabwe. At 6 months after intervention initiation, participants in clinics randomized to the FB had fewer common mental disorders and depressive and anxiety symptoms, lower disability severity, and reduced suicidal ideation compared to those randomized to augmented usual care.^44^ Further, participants with suicidal ideation who received the FB experienced similar reductions in symptoms of common mental disorders as those without,^20^ suggesting the FB problem-solving may already support a reduction in SIBs.

# METHODOLOGY

## Design of Study

In this randomized controlled pilot trial across five public health clinics, participants will be individually randomized 1:1 either to enhanced FB+SP (N = 30) or augmented usual care (N = 30) and followed for 6 months. Primary and secondary outcomes will be assessed at 2-, 4-, and 6-week; and 3- and 6- month study visits and compared across the two study arms.

**Box 1: FB+SP Package**

Session 1 (Week 1; 45-60 min)

- SIBs & Suicide Risk Assessment

- Psychoeducation & FB+SP Introduction

- SP activities & Strategy Creation

Sessions 2 (Week 2; 40-45 min)

- SIBs & Suicide Risk Assessment

- SP Check-in & Revision

- FB Problem-Solving Initiation

Sessions 3-6 (Weeks 3-6; 15-20 min)

- SIBs & Suicide Risk Assessment

- SP Check-in & Revision

- FB Problem-Solving Continuation

Concurrent Peer-Support

- 6 group peer-support sessions

- Delivered Monthly

*Optional Ad-hoc Support*

- Telephone/text message check-ins

FB+SP: The FB+SP protocol will embed SP within the existing adolescent-friendly FB model, tailored for the Malawian context.^22,53^ The protocol will include six sessions, starting with the development of the SP during the first session (**Box 1**). Each of the following sessions will include SIBs and suicide risk assessments, SP check-ins and revisions, as well as FB problem-solving to address SIBs and suicide risk (**Box 1**). SP+FB will be delivered by selection of young counselors (mixed genders, aged 20-35) who are motivated to work with young people. Counselors will be trained to deliver non-judgmental counseling that respects the privacy and autonomy of ALWH and enables them to make free and informed choices that are relevant to their individual needs. Training will also include discussion of adolescent development and the unique experiences of ALWH related to stigma, disclosure, social relationship issues, and health care access. Counseling sessions will take place in a youth oriented but discrete location within the HIV clinic and be available on weekends to ensure accessibility.

SP will be initiated on the first session and will consist of an introduction to the program, a SIBs and suicide risk narrative assessment and the co-development of a personalized list of coping strategies. SIBs and suicide risk will continue to be assessed at follow-up sessions, with option telephone and/or text message check-ins.

FB problem-solving counseling will begin on the second session. The first FB session includes three components called Opening the Mind, Uplifting, and Strengthening, with subsequent sessions building on the first. Opening the Mind refers to the therapeutic process by which, through asking questions, clients are encouraged to open their minds to identify their problems, choose one to work on, identify a feasible solution, and agree on an action plan through an iterative process guided by the counselor. Each structured session lasts 30-45 minutes and will be conducted in a private clinic room in the participant’s local language (Chichewa). No specific retention support will be provided, but participants may identify barriers to engagement in HIV care to address during their counseling session. After 4 sessions of individual therapy, the counselor can refer participants not improving or with suicidal ideation to a supervisor trained in mental health to reassess and manage the case. Case management may include additional counseling or pharmacotherapy, at the discretion of the managing clinician.

Additionally, adolescents will be offered structure peer support via in person group meetings to facilitate engagement in care for ALWH and support SIBS reduction, as is now standard with the existing adolescent-FB package.^53^ Peer supporters will be mature young adults (aged 18-21) who have completed secondary school, are openly living with HIV, and motivated to work with adolescents. The counselors must be willing to maintain participant and colleague confidentiality and sign a confidentiality agreement. Peer Supporters will be trained to deliver youth-friendly group sessions related to HIV care. The content of the 6 peer support sessions includes: Mental Health and HIV, Status Communication, Adherence and Viral Load Testing, Secondary Prevention (Sex and Relationships), Stigma, and Planning for the Future. The 6 sessions will be delivered monthly over 6 months and last 90 minutes each. The group sessions will be in a private space in the clinic in the participant’s local language (Chichewa).

Augmented usual care: Care for suicidality in public facilities in Malawi includes options for basic supportive counseling by the primary provider or nurse, medication management by the primary provider, referral to the clinic’s psychiatric nurse, or, for acute cases or crises, referral to the psychiatric units at tertiary care hospitals (Bwaila Hospital in Lilongwe District). Nurses and clinicians at the study sites have been specifically trained to use the Tool for Assessment of Suicide Risk for Adolescents (TASR-A) to assess ALWH considered at elevated risk for suicide.^54,55^ For this study, usual care will be augmented by a trained study nurse who will provide mental health evaluation, brief supportive counseling, information, education and support on SIBs, and (if indicated) facilitation of referral to the clinic’s psychiatric nurse or to Bwaila Hospital. The study nurse will have up to 3 follow-up contacts with the participant to assess whether they have followed up on recommended referrals or treatment plans and to assess whether any further outreach is needed.

## Place of study

The study will be conducted at five public clinics in Lilongwe, Malawi: Area 18, Area 25, Lighthouse, Kawale, and Likuni Health Centers.

## Target Population

ALWH: We will recruit ALWH (N = 60) from the five study clinics in Lilongwe (N = 12 per site), Malawi who will be screened with the combined Patient Health Questionnaire-9 modified for adolescents (PHQ-9-A)^51^ and Ask Suicide-Screening Questions (ASQ).^56^ We will aim to recruit for diversity in age, gender identity, and HIV acquisition type. ALWH will be eligible for the study if they are: (1) age 13-19; (2) diagnosed with HIV; (3) report current or historical SIBs on the PHQ-9-A/ASQ;^50,56^ (4) living in the clinic’s catchment area with intention to remain for > 1 year; and (5) willing to provide consent (age 18+ or 16-17 years old and married and thereby considered emancipated minors per Malawi law) or assent with parental consent (age 13-17). All ALWH will be eligible for recruitment including ART initiators, ART re-initiators and established patients. ALWH reporting SIBs who choose not to participate will be referred to a mental health provider. A random sample of 10 ALWH participants will be invited to participate in exit interviews to assess intervention delivery and contextual factors that impeded and facilitated implementation.

Clinical Staff: We will conduct exit interviews with a random sample of clinical staff (N = 10) involved in the delivery and/or supervision of the FB+SP intervention.

## Sampling Techniques and Tools

Research assistants will work with clinical staff to screen and recruit ALWH from five HIV clinics in Lilongwe, Malawi. A random sample of 10 ALWH participants and a random sample 10 clinic staff involved in the delivery and/or supervision of FB+SP will be invited to participate in exit interviews to assess intervention delivery and contextual factors that impeded and facilitated implementation.

## Sample Size Determination

Trial: We expect to be able to recruit 60 ALWH. The primary goal of this R34 proposal is to evaluate the feasibility, acceptability, and fidelity of the enhanced FB+SP model to address suicidality ALWH. In Aim 2, we will enroll 60 ALWH with SIBs in a 2-arm randomized pilot (30 per arm) to compare augmented usual care with the enhanced FB+SP intervention. Information on preliminary effectiveness of the intervention across a range of mental health outcomes related to SIBs will be assessed, but interpreted cautiously as efficacy is only a secondary aim of this pilot study. Intervention effectiveness will subsequently be assessed in a large-scale R01 clinic-level RCT. The sample of 60 ALWH (30 per arm) will be sufficient to estimate quantitative measures of feasibility and acceptability with reasonable precision (e.g., standard errors of .06-.09 for proportions).

Qualitative Interviews: Some providers involved in the delivery of the FB+SP intervention (N=10) and patients in the intervention arm (N=10) will be asked to complete brief exit interviews at their study sites to assess facilitators and barriers to the delivery and receipt of the FB+SP intervention. As is common in qualitative research, we aim to continue data collection until data saturation has been achieved. Based on prior experiences employing these methods and achieving saturation, this sample size is expected to be sufficient to capture a diverse range of perspectives as well as achieve theme saturation in each group.

## Data Collection Techniques and Tool

Implementation outcomes: This trial will explore patient and provider ***acceptability*** and ascertain ***feasibility*** of recruitment, retention, and participation. We will conduct audio-recorded semi-structured qualitative interviews with a random sample of patients enrolled in the study (N = 10). Exit interviews will explore acceptability of the intervention to patients (ease of participation or delivery, perceived usefulness of the intervention, suggestions for improvement, and contextual factors that impeded or facilitated implementation). Acceptability will also be assessed quantitatively with the *Client Satisfaction Questionnaire-8*.^57^ Feasibility will be assessed through the number of ALWH enrolled; a comparison of planned relative to actual enrollment; reasons for non-enrollment; the proportion of ALWH retained in each arm; and the number of sessions attended during the study period. Additionally, we will interview clinic staff (N = 10) involved in the delivery and/or supervision of the intervention to assess acceptability to providers and feasibility (perceived changes, comfort level and competency, training satisfaction, recommendations, barriers and facilitators). We will monitor ***fidelity*** to the intervention protocol using the *Fidelity Checklist­* and in-depth interviews with clinic staff (**Table 1**).

Preliminary Effectiveness: This trial will assess patient outcomes as a measure of preliminary effectiveness, comparing the prevalence of SIBs, suicide risk, and depressive symptoms between the two study arms. Patients will be surveyed by the RAs at enrollment (baseline), 2 weeks post-enrollment (FU1), 4 weeks post-enrollment (FU2), 6 weeks post enrollment (FU3), 3 months post-enrollment (FU4), and 6 months post-enrollment (FU5) (**Table 1**). VL draws will occur at enrollment (baseline) and 6 months post-enrollment (FU5). Additionally, RS will abstract clinical data related to ART appointment attendants, providers assessment of patients’ depressive symptom and suicidal ideation and behaviors and continue treatment as clinically indicated.

| **Table 1. Assessment Domains, Constructs, and Measures** | | | | |
| --- | --- | --- | --- | --- |
| **Domain** | **Construct** | **Measures** | **Assessment Point** | **Data**  **Type** |
| **Primary Implementation Outcomes** | | | | |
|  | Feasibility | # enrolled; # planned vs. # actual enrollment; reasons for non-enrollment; % retained in each arm; # of sessions attended | Ongoing | Clinic |
|  |  | In-depth interviews | Exit Interview | Qual. |
|  | Fidelity | Checklist for content covered during sessions | Ongoing | Clinic |
|  | Acceptability | Adapted Client Satisfaction Questionnaire-8^57^ | FU1, FU2 | Survey |
|  |  | In-depth interviews | Exit Interview | Qual. |
| **Secondary Preliminary Efficacy** | | | | |
|  | SIBs | PHQ-9-A Question 9 and ASQ Tool^50,56^ | Ongoing | Clinic |
|  | Suicide Risk | Suicide Risk Assessment Protocol^17^ | Ongoing | Clinic |
|  | Depressive Symptoms | PHQ-9-A^50^ | Ongoing | Clinic |
| **Covariates & Mediators** | | | | |
|  | Demographics | Age, sex, resources, education, religion, occupation, health status, awareness of HIV status at ART initiation, home distance | BL | Survey |
|  | Disclosure | HIV and Suicidality Disclosure to Family and/or Friends | BL | Survey |
|  | MH Care Engagement | FB+SP appointment dates, other MH treatment | Ongoing | Clinic |
|  | HIV Care Engagement | ART appointment dates, ART pill count, VL draws | Ongoing | Clinic |
|  |  | VL draws | BL, FU5 | Study |
|  | HIV Stigma | Internalized AIDS-Related Stigma Scale^58^ | BL, FU3-5 | Survey |
|  | Suicide Stigma | Personalized Suicide Stigma Questionnaire^59^ | FU3-5 | Survey |
|  |  |  |  |  |
|  | Suicide Coping | Suicide Related Coping Scale^60^ | BL, FU3-5 | Survey |
|  | Isolation | Adapted Interpersonal Needs Questionnaire items^61^ | BL, FU3-5 | Survey |
|  | Burdensomeness | Adapted-Suicide Cognitions Subscale items^62^ | BL, FU3-5 | Survey |
|  | Hopelessness | Adapted-Beck Hopelessness Scale items^63,64^ | BL, FU3-5 | Survey |
|  | Acquired Capability | Adapted-Acquired Capability for Suicide Scale items^61^ | BL, FU3-5 | Survey |
|  | Sleep Quality | Pittsburgh Sleep Quality Index^65^ | BL, FU3-5 | Survey |
|  | Trauma | PC-PTSD-5^66^ | BL, FU3-5 | Survey |
|  | Alcohol/Marijuana | Single-question alcohol screening test^67^; single-item screen-cannabis.^68^ | BL, FU3-5 | Survey |
|  | Social Support | Multi-dimensional Scale of Perceived Social Support^69,70^ | BL, FU3-5 | Survey |
|  | Resilience | PLHIV Resilience Scale^71^ | BL, FU3-5 | Survey |
| **NIH Common Data Elements** | | |  |  |
|  | Anxiety, Depression, Functioning | Revised Children’s Anxiety^72^ and Depression Scale-25 (RCADS-25) Youth Assessment^73^ | BL, FU3-5 | Survey |
|  | DSM-5 Cross-cutting | DSM-5 Cross-cutting assessment Youth Self Report ^74,75^ | BL, FU3-5 | Survey |

## Data Analysis

Baseline characteristics of participants will be compared between the arms using t-tests for continuous variables and chi-square tests for categorical variables to assess balance across study arms.

Primary Implementation Outcomes: Quantitative measures of feasibility, acceptability, and fidelity will be summarized using means and standard deviations or proportions and compared across arms using statistical models for continuous or binary outcomes, as appropriate. Specifically, we will compare the mean number of people enrolled and the proportion retained in each arm through 6 months (feasibility); the proportion of participants who found the intervention easy to administer and helpful (acceptability), and the proportion of interventionists covering at least 80% of checklist items during random direct monitoring sessions (fidelity). Qualitative measures of acceptability (e.g. open-ended questions on the exit interview) will be analyzed using textual data analysis steps described in Aim 1 (reading for content; coding; data display, data reduction and interpretation).^76,77^

Secondary Preliminary Efficacy: Preliminary efficacy will be analyzed at 2, 4, and 6 weeks and 3 and 6 months and compared between study arms. In addition, statistical models examining differences in prevalence of SIBs, suicide risk, and depressive symptoms will account for baseline values by using generalized estimating equations with an exchangeable correlation matrix. Importantly for the design of the subsequent RCT, this pilot will yield estimates of the within-clinic intra-cluster correlation and the standard deviation for the effectiveness outcomes.

## Dissemination of Results

Local dissemination of evaluation results is critical to the success of this project. The goal is to publish all results and disseminate them at national and international scientific meetings. Results will also be distributed freely to colleagues across Malawi involved in the care of ALWH. This will include presentations to be given at UNC Project Malawi, the Malawi Ministry of Health Technical Working Groups, Kamuzu University of Health Sciences dissemination meetings, NHSRC meetings and through the UNCPM community advisory board. We will also present results at national and international conferences (e.g., IAS, CROI, Academy Health’s annual Conference on the Science of Dissemination and Implementation, the International Workshop on HIV & Adolescence, International Conference on HIV Treatment and Prevention Adherence). We will disseminate our results with the NHSRC. Abstracts presented at conferences will also be prepared for publication to disseminate results to the wider scientific community.

# ETHICAL CONCERNS

## Human Subjects

Safety considerations: Study staff will evaluate patients for safety who endorse any level of suicidal ideation whether or not they enroll in the study. Safety assessment results will be conveyed to the clinical team as appropriate for further follow-up according to the clinic’s standard operating procedures.

## Institutional Review Board

Prior to implementation of the formative research, all protocol materials will be reviewed by the University of Pennsylvania Institutional Review Board, in the U.S., and the Malawi National Health Sciences Research Committee (NHSRC) before any data collection or analysis occurs. The study will also be reviewed and informed by the existing community advisory board (CAB) of UNC Project in Malawi. The CAB reviewed first reviewed the study in July 2024. The CAB will provide ongoing review, providing feedback on the protocol and implementation. All research procedures will adhere to Malawian and US ethical standards for research involving human subjects.

## Confidentiality, Risks, and Risk Minimization

Risks of loss of confidentiality or social harms to participants will be minimized by 1) training of study staff in the ethical conduct of research; 2) strict protection of confidentiality and personal information; 3) close monitoring of social harms with appropriate IRB reporting.

All study data will be kept in a locked cabinet at UNC Project, where they will be maintained in a locked office accessible only to study staff. All data will be de-identified before analysis. Only the principal investigator, data entry personnel, and assigned data analysts will have access to the de-identified electronic study database. All study data will be destroyed 1 year after the completion of all study-related activities.

Discussing personal information: Data collection will include asking ALWH about their mental health. All data associates will be trained to keep all information confidential. Research assistants will be trained, and participants informed, that they can pause or discontinue a research interview at any time if they find the topic upsetting.

Data security: All study data, including interview guides, audio recordings of interviews, interview transcribes, and logbooks will be kept in a locked cabinet at the UNC Project data center, where they will be maintained in a locked office at UNC Project accessible only to the principal investigator. Interviews that are transcribed and computerized will be stored on the secure central server at UNC Project. For analysis purposes, data will be de-identified before coding and analysis. Only the principal investigator, data entry personnel, and assigned data analysts will have access to the de-identified electronic study database.

Preventing Discomfort: The surveys, counseling sessions, and peer support activities may cause discomfort for some participants. Efforts will be made to minimize this discomfort by assuring that participants are informed beforehand about the nature of the interaction and that the interaction is completed in a private setting. Participants will be informed that they have the right to decline participation in the study, to refuse to answer any questions, or to withdraw at any time. The consent form will also emphasize that personal information (and inclusion in the study based on HIV and suicidality) will be shared during focus groups and confidentiality cannot be assured.

Addressing Harms: Our overall goal is to protect the well-being of ALWH, thus if sexual or physical abuse is reported, we will ensure ALWH are referred to a local organization that deals with sexual abuse/violence so that the individual receives appropriate care or to a local social worker. If a study team member comes to believe that a participant is experiencing a crisis (which the participant may or may not recognize) they also may refer to local social workers for counseling or psychosocial support. Examples would include a participant who is having an extreme emotional reaction when discussing the challenges of living with HIV, or a participant whose behavior suggests possible undiagnosed mental illness. These revelations of harm are not common but may occur on a limited basis, necessitating that study protocols be in place to respond appropriately.

At each clinic from which we will recruit participants, we will identify a provider to act as the “clinic lead” for navigation services. This person is a chief point of contact for the study team. When a situation arises where study staff learns of a potential risk of harm to a participant, the following steps will be followed:

1. The study team member learning of the problem will immediately inform the PI.
2. If the participant is actively in distress and at the clinic, the study team member will refer the participant to the clinic lead (or, if the lead is not available, to another clinic provider) for immediate evaluation.
3. If the participant is actively in distress and not at the health center, the study team member will offer to meet the participant and accompanying them to the health facility for immediate referral.
4. If the participant is not actively in distress, then the study investigators, project manager, and other appropriate project supervisory staff will develop a plan for referring the participant to the clinic lead. As appropriate, the project team may consult the clinic lead in developing the plan. (All consent forms will note that investigators may need to break confidentiality when there is a threat of harm to self or others.) The timing and most appropriate means of effecting a referral will be dependent on the nature of the problem. For example, if a participant reports child abuse in the home, then it would be necessary to involve appropriate social services to investigate the problem. By contrast, if a participant reports that she/he is facing ongoing (but not life threatening) emotional abuse from a partner, then it may make more sense for the navigator to first approach the participant and encourage her/him to meet with a clinic provider or with other social support services in the area.
5. Study investigators will inform all governing IRBs of the incident and update the IRBs as necessary on the outcomes of efforts to intercede and resolve the harm.

Response to suicidal thoughts, ideation or behaviors: For participants who indicate any SIBs during research assessments, IDIs or FGDs, we will follow a suicide risk assessment and response protocol that we have previously deployed in several prior studies in Malawi.^17^ Using this structured protocol, the research assistant will assess suicide risk using the Suicide Risk Assessment Protocol (SRAP) questions.^17^ These questions will allow for differentiation between passive and active suicidal thoughts and assessment of suicide risk factors to further classify active suicidal thoughts into low, medium, or high risk. All participants reporting SIBs will see a clinician that same day. Following the protocol, the research staff will accompany the participant to a clinician with the results of the risk assessment for the clinician to then decide on appropriate further clinical response following the clinic’s standard safety procedures. All clinical staff at the study sites have been trained on suicide risk management, and are able to provide supportive counseling, review a safety plan, provide medication management, and refer to the clinic’s psychiatric nurse as necessary. For acute cases or crises, all clinic staff are trained to refer to the psychiatric unit at Bwaila Hospital in Lilongwe District. Additionally, we will train a study nurse who will be available on-site to assist the existing clinical staff, provide mental health evaluation, brief supportive counseling, information, education and support on SIBs as needed. For acute cases or crises, the study nurse will facilitate referral to the psychiatric unit at Bwaila Hospital. The study nurse will have up to 3 follow-up contacts with all participants reporting SIBs to assess whether they have followed up on recommended referrals or treatment plans and to assess whether any further outreach is needed.

Response intimate partner violence: For enrolled participants who indicate any recent sexual or physical violence by an intimate partner on the participant survey section on intimate partner violence or who otherwise indicate a potentially unsafe home situation, the study team will follow a IPV safety assessment and response protocol that we have deployed successfully in prior studies in Malawi. All participating health facilities have a One-Stop Centre with one or more behavioral health professionals on staff who can discuss reporting and support options with an affected individual. In Malawi, reports of intimate partner violence are handled by a designated Victim Support Unit of the local police department. Our safety protocol guides our study team member to discuss with the participant the available support services and options, including going to the facility’s One Stop Centre to meet with a behavioral health professional and connecting with the local police department’s Victim Support Unit. Whether the participant takes any action will be left at the discretion of the participant since reporting can carry its own risks. When possible, the study team member will provide a warm handoff to a facility clinician to further discuss the participant’s situation and options. If the participant chooses not to meet with a clinician, the study team member will ask the participant to sign the study safety assessment form indicating that they are declining to meet with a clinician.

## Benefits to Participants

This study has some minimal risks associated with participation and we anticipate that few participants will experience negative events as a result of taking part in the study. This research will improve our understanding of how the enhanced FB-SP model may address SIBs among ALWH. Long-term, supporting ALWH with SIBs through a counseling intervention carried out by counselors is expected to reduce SIBs, improve engagement in HIV-care, and ultimately improve adolescent health and HIV outcomes. Therefore, the risk to individual participants in our study is small and the potential benefit to society is substantial.

## Costs and Compensation

Study participants will receive standard compensation in line with National Health Services Research Committee (NHSRC) and UNC Project Malawi practices for their enrollment visit and research outcome interviews. Namely, participants will receive a travel reimbursement equivalent to 10USD (~17000MK) for their participation.

## Informed Consent

In this study, we are seeking to enroll ALWH 13-19 years to participate. ALWH who are 18 years or older will provide consent for themselves. ALWH 13-17 years will provide assent and will have an authorized adult over 18 years provide consent for them prior to participation. The authorized adult can be a parent, a legal guardian, or someone designated as an authorized representative. The research study will be described briefly to all adolescents accessing ART services at Area 18, Area 25, or Kawale Health Centers in Lilongwe while they wait in the reception area. Once a participant is determined to be eligible for the study a trained interviewer will complete an informed consent process with each participant in their native language, Chichewa. During the informed consent process, the counselor will describe the procedures to be followed, the risks and benefits of participation, the duration of participation, and the steps taken to protect participant’s confidentiality, particularly with respect to keeping all personal and private information (such as HIV, depression status, and SIBs) private to the extent possible by the study team. The counselor will also emphasize that the consent form says that confidentiality of sensitive information cannot be assured during focus groups. Illiterate participants may sign the consent form via thumbprint, in the presence of an impartial witness. Any questions or concerns about privacy will be answered by the counselor or referred to the PI, who will address them. In order to provide protection to this group, we will have rigorous trainings of research staff. We will teach staff to be sure that there is an adequate assessment of understanding. Staff will ensure participants fully understand the content, and time for questions will be encouraged, and answered honestly. We will be clear that refusals will not compromise access to services in any way. We will follow a work practice guideline of when mandatory reporting is required and the best methods to do so, e.g. evidence of exploitation or sexual abuse. Additionally, both sites will adequately map out referrals services to deal with a range of potential needs. All ALWH who screen positive for depression or SIBs will be referred to outpatient psychiatry services that are currently available and staffed by master’s level nursing staff that can prescribe antidepressant medications. ALWH reporting SIBs who choose not to participate will be referred to a mental health provider. Written informed consent will be obtained from each participant and participants will be provided with a copy of their informed consent forms if they are willing to receive it. Study staff will document the informed consent process.

## Adverse Event Reporting

We do not anticipate any adverse events occurring given the nature of the minimal risks associated with this research. However, in the case that a breach of confidentiality does occur, the study team will immediately inform the IRBs. Specifically:

- Deaths related to study participation shall be reported by the PI to the NIMH program officer (PO) immediately and no later than within 5 business days of the PI first learning of the death
- Serious Adverse Events (SAEs) related to study participation shall be reported by the PI to the NIMH PO within 10 business days of the study team becoming aware of the SAE
- Unanticipated Problems Involving Risks to Subjects or Others shall be reported by the PI to the NIMH PO within 10 business days of the study team becoming aware of the problem
- Adverse events and SAEs, including deaths, that are deemed expected and/or unrelated to the study shall be submitted in summary form to the NIMH PO with the annual progress report
- All reported social harms will be documented and communicated to NHSRC and UNC IRB in 7 days
- Protocol violations shall be submitted in summary form to the NIMH PO with the annual progress report
- Suspension or termination of study by IRB shall be reported by the PI to the NIMH PO within 3 business days of receipt

## Study Discontinuation

The study may be discontinued at any time by the University of Pennsylvania IRB, the NHSRC, or other government agencies as part of their duties to ensure that research participants are protected.

## Amendments to Protocol

Should amendments to the protocol be required, the amendments will be originated and documented by the Principal Investigator at UNC and NHSRC. It should also be noted that when an amendment to the protocol substantially alters the study design or the potential risk to the patient, a revised consent form might be required. The written amendment, and if required the amended consent form, must be sent to UNC’s IRB and NHSRC for approval prior to implementation.

## Record Retention

Study documentation includes all report Forms, data collection forms, source documents, Sponsor-Investigator correspondence, and regulatory documents (e.g., protocol and amendments, IRB correspondence and approval, signed patient consent forms). These documents will be retained for 3 years after the completion and final study report.

# PERSONNEL ROLES AND INSTITUTIONS

**Principal Investigators**

Kazione Kulisewa, MBBS MMed

Malawi College of Medicine

No 1 Mahatma Ghandi Road

Blantyre, Malawi

Phone: +265 1871911

Email: kkulisewa@yahoo.com

Melissa Stockton, PhD, MSPH

Perelman School of Medicine

University of Pennsylvania

3535 Market St.

Philadelphia, PA 19104, USA

Phone: 0980105758; +141903407476

Email: melissaann.stockton@pennmedicine.upenn.edu

**Co-Investigators**

Dr. James January

Kamuzu University of Health Sciences

No 1 Mahatma Ghandi Road

Blantyre, Malawi

Phone: +265 1871911

Email: jamesjanuary4@gmail.com

Dr. Charles Masulani-Mwale

St. John of God Hospitaller Services

Katoto, Off Karonga Road, Box 744

Mzuzu, Malawi

Phone: +265 9999 27938

Email: charles.masulani@sjog.mw

Bradley Gaynes, MD

UNC School of Medicine

Department of Psychiatry

CH# 7160, 130 Mason Farm Rd

Chapel Hill, NC 27599 USA

Phone: 919-445-0214

Email: bradley_gaynes@med.unc.edu

Michael Udedi, PhD

Malawi Ministry of Health P. O. Box 3037

Lilongwe, Malawi

Phone: + 265 1 789 400

Email: mphatsoudedi@yahoo.co.uk

Nivedita Bhushan, PhD

RTI International

Center for Communication Science

3040 E. Cornwallis Road

Research Triangle Park, NC 27709 USA

Phone: 919-248-8509

Email: nbhushan@rti.org

Dr. Gregory Brown

University of Pennsylvania

Penn Center for the Prevention of Suicide

3535 Market St

Philadelphia, PA 19104, USA

Phone: 215-898-4104

Email: [gregbrow@pennmedicine.upenn.edu](mailto:gregbrow@pennmedicine.upenn.edu)

# 7.0 WORKPLAN

This pilot trial will be conducted over 18 months. Psychosocial Counselor and Peer Supporter trainings will be conducted in the first 2 months. The pilot trial will be conducted over 12 months. All activities will launch following regulatory approvals.

|  | 2025 | | | | | | | 2026 | | | | | | | | | | |
| --- | --- | --- | --- | --- | --- | --- | --- | --- | --- | --- | --- | --- | --- | --- | --- | --- | --- | --- |
| Calendar Month | A | M | J | J | A | S | O | V | D | J | F | M | A | M | J | J | A | S |
| # Month of Project Period | 1 | 2 | 3 | 4 | 5 | 6 | 7 | 8 | 9 | 10 | 11 | 12 | 13 | 14 | 15 | 16 | 17 | 18 |
| Psychosocial Counselor &  Peer Supporter Training |  |  |  |  |  |  |  |  |  |  |  |  |  |  |  |  |  |  |
| Pilot Implementation and Follow-up |  |  |  |  |  |  |  |  |  |  |  |  |  |  |  |  |  |  |
| Pilot Results Analysis and Resulting Manuscript |  |  |  |  |  |  |  |  |  |  |  |  |  |  |  |  |  |  |

# BUDGET

| **Line Item** | **Cost per unit** | **# and type units** | **Cost**  **(USD)** | **Cost (Kwacha)** |
| --- | --- | --- | --- | --- |
| **1. Study Staff** |  |  |  |  |
| Research Assistant | $450 | 2 x 12 months | $10,800.00 | 18,910,800.00 |
| Half-time Research Nurse (SIB management support) following research visits | $450 | 1 x 6 months | $2,700.00 | 4,727,700.00 |
| **2. Compensation for study participants and guardians** | | | | |
| Travel reimbursement for research for participants | $10 | 6 study visits x 60 participants | $3,600.00 | 6,303,600.00 |
| Travel reimbursement for enrollment visit for guardians | $10 | 1 enrollment visit x 60 guardians | $600.00 | 1,050,600.00 |
| Travel reimbursement for counseling & peer support sessions | $5 | 360 Sessions | $1,800.00 | 3,151,800.00 |
| Travel reimbursement for exit interviews | $10 | 10 ALWH & 10 Staff | $200.00 | 350,200.00 |
| **3. Other Research Costs** | | | | |
| HIV Testing | $45 | 2x60 Participants | $5,400.00 | 9,455,400.00 |
| Translation Fees |  |  | $200.00 | 350,200.00 |
| Malawi IRB Fee |  |  | $150.00 | 262,650.00 |
|  | | | | |
| **Total** |  |  | **$25,450.00** | 44,562,950.00 |
| **10% NHSRC fee** |  |  | **$2,545.00** | 4,456,295.00 |
| **GRAND TOTAL (fee included)** |  |  | **$27,995.00** | 49,019,245.00 |

## Budget Justification (USD)

Personnel

Two Research Assistants in Malawi ($10,800 - 100% effort for 12.0 months) – Two research assistants in Malawi will be hired and trained to assist with the pilot trial.  $450 salary/month x 12 months x 2 research assistants = $10,800

Additionally, we will hire a research nurse half-time to support SIBs management and provide follow-up to ensure appropriate referrals and/or treatment have been implemented. $450 salary/month @ 50% effort x 6 months = $2,700

Total Personnel Costs $ 13,500.

Compensation for Study Participants and Guardians

Travel Reimbursement for study visits for participants ($3,600) – A total of 60 participants will be included in our proposed aims. Each participant will receive $10 per study visit (6 per participant) for travel reimbursement.

Travel Reimbursement for enrollment visit for guardians ($600) – Each guardian (for 60 adolescent participants aged 13-19) will receive $10 for travel reimbursement for the initial enrollment visit.

Travel Reimbursement for counseling/peer support sessions for participants ($1,800) – Each participant will receive $5 for travel reimbursement for each counseling or peer support session. 30 participants will receive 6 counseling sessions and 6 peer support sessions (12 sessions total).

Travel Reimbursement for exit interviews ($200) – Each participant will receive $10 for travel reimbursement for each exit interview. 10 adolescent participants and 10 healthcare facility staff will participate in the exit interviews.

Total Compensation for Study Participants and Guardians Costs $ 6,200.00

OTHER RESEARCH COSTS

HIV Testing ($5400) – We will collect viral loads at 45 USD per test for all 60 participants at enrollment and 6 months.

Translation and Transcription Fees ($200) – All study materials including informed consent forms, surveys, and interview guides will be translated into Chichewa, the local language in Lilongwe, Malawi. Translation fees $200.

Malawi IRB Fee ($150) – All research in Malawi must receive ethical approval from the National Health Science Research Committee, which charges a $150 fee to review all applications for ethical approval.

Total Other Research Costs $350

**TOTAL COSTS: $25,450.00 / 44,562,950.00 MWK**

**10% NHSRC Fee: $2,545.00 / 4,456,295.00 MWK**

**GRAND TOTAL (Inclusive of 10% NHSRC fee): $ 27,995.00 / MWK 49,019,245.00**

# REFERENCES

1. Roth GA, Abate D, Abate KH. Global, regional, and national age-sex-specific mortality for 282 causes of death in 195 countries and territories, 1980-2017: a systematic analysis for the Global Burden of Disease Study 2017. *The Lancet*. 2018;392(10159):1736-1788.

2. Mokdad AH, Mohammad HF, Farah D, al. e. Global burden of diseases, injuries, and risk factors for young people's health during 1990-2013: a systematic analysis for the Global Burden of Disease Study 2013. *The Lancet*. 2016;387(10036):2383-401. doi:10.1016/S0140-6736(16)00648-6.

3. Quarshie ENB, Waterman MG, House AO. Self-harm with suicidal and non-suicidal intent in young people in sub-Saharan Africa: a systematic review. *BMC Psychiatry*. 2020;20(234)

4. Bantjes J, lemmi V, Coast E, et al. Poverty and suicide research in low- and middle- income countries: systematic mapping of literature published in English and proposed research agenda. *Global Mental Health (Cambridge)*. 2016;13:e32. doi:10.1017/gmh.2016.27

5. Arsenault-Lapierre G, Kim C, Turecki G. Psychiatric diagnoses in 3275 suicides: a meta-analysis. *BMC Psychiatry*. 2004;4(4):37. doi:10.1186/1471-244X-4-37

6. Fleischmann A, Bertolote JM, Belfer M, Beautrais A. Completed suicide and psychiatric diagnoses in young people: a critical examination of the evidence. *Am J Orthopsychiatry*. 2005;75:676-683.

7. Knipe D, Williams JA, Hannam-Swain S, et al. Psychiatric morbidity and suicidal behaviour in low- and middle-income countries: A systematic review and meta-analysis. *PLoS One Med*. 2019;16:e1002905. doi:10.1371/journal.pmed.1002905.

8. MacLean SA, Lancaster KE, Lungu T, et al. Prevalence and correlates of probable depression and post-traumatic stress disorder among female sex workers in Lilongwe, Malawi. *International Jounral of Mental Health and Addiction*. 2018;16(1):150-163. doi:10.1007/s11469-017-9829-9.

9. Malava JK. Prevalence and correlates of probable depression diagnosis and suicidal ideation among patients receiving HIV care in Lilongwe, Malawi. *Malawi Medical Journal*. 2018;30(4):236-242.

10. Pengpid S, Peltzer K. Prevalence and correlates of suicidal behaviour among adults in Malawi: a nationally representative cross-sectional survey in 2017. *International journal of mental health systems*. 2021;15:1-8.

11. Shaikh MA, Lloyd J, Acquah E, Celedonia KL, Wilson ML. Suicide attempts and behavioral correlates among a nationally representative sample of school-attending adolescents in the Republic of Malawi. *BMC Public Health*. 2016;16

12. Casale M, Boyes M, Pantelic M, Toska E, Cluver. Suicidal thoughts and behaviour among South African adolescents living with HIV: Can social support buffer the impact of stigma? *Journal of Affective Disorders*. 2019;245:82-90.

13. Suicide Prevention Implementation Framework (2023).

14. Wu KC-C, Cai Z, Chang Q, Chang S-S, Yip PSF, Chen Y-Y. Criminalisation of suicide and suicide rates: an ecological study of 171 countries in the world. *Bmj open*. 2022;12(2):e049425.

15. Southern Africa Litigation Centre. Malawi: Man arrested and convicted for attempting to commit suicide. Southern Africa Litigation Centre. 2023. <https://www.southernafricalitigationcentre.org/2022/11/10/malawi-man-arrested-and-convicted-for-attempting-to-commit-suicide/>

16. Crabb J. Attitudes towards mental illness in Malawi: a cross-sectional survey. *BMC Public Health*. 2012;12(541)

17. Landrum KR, Akiba CF, Pence BW, et al. Assessing suicidality during the SARS-CoV-2 pandemic: Lessons learned from adaptation and implementation of a telephone-based suicide risk assessment and response protocol in Malawi. *Plos one*. 2023;18(3):e0281711.

18. Chibanda D, Mesu P, Kajawu L, Cowan F, Araya R, Abas MA. Problem-solving therapy for depression and common mental disorders in Zimbabwe: piloting a task-shifting primary mental health care intervention in a population with a high prevalence of people living with HIV. *BMC public health*. 2011;11(1):828.

19. Pierce D. Problem solving therapy - use and effectiveness in general practice. *Aust Fam Physician*. 2012;41:676-679.

20. Munetsi E, Simms V, Dzapasi L, et al. Trained lay health workers reduce common mental disorder symptoms of adults with suicidal ideation in Zimbabwe: a cohort study. *BMC public health*. 2018;18:1-7.

21. Nuji C, van Ballegooijen W, De Beurs D, al. e. Safety planning-type interventions for suicide prevention: meta-analysis. *The British Journal of Psychiatry*. 2021;219(2):419-426.

22. Stanley B, Brown GK. Safety planning intervention: a brief intervention to mitigate suicide risk. *Cognitive and behavioral practice*. 2012;19(2):256-264.

23. Knettel BA, Knippler E, Martinez A, et al. A scoping review of counseling interventions for suicide prevention in Africa: Few studies address this life-saving aspect of mental health treatment. *Journal of affective disorders*. 2023;

24. Hoeft JT, Fortney CJ, Patel V, Unützer J. Task-Sharing Approaches to Improve Mental Health Care in Rural and Other Low-Resource Settings: A Systematic Review. *J Rural Health*. 2018;34:48-62. doi:10.1111/jrh.12229.

25. Wainberg ML, Gouveia ML, Stockton MA, et al. Technology and implementation science to forge the future of evidence-based psychotherapies: the PRIDE scale-up study. *Evidence-based mental health*. 2021;24(1):19-24.

26. Udedi M, Stockton MA, Kulisewa K, et al. Integrating depression management into HIV primary care in central Malawi: the implementation of a pilot capacity building program. *BMC health services research*. 2018;18(1):593.

27. Kulisewa K, Dussault JM, Gaynes BN, et al. The feasibility and acceptability of a task-shifted intervention for perinatal depression among women living with HIV in Malawi: a qualitative analysis. *BMC psychiatry*. 2022;22(1):1-13.

28. Burgess AR. Supporting mental health in South African HIV-affected communities: primary health care professionals’ understandings and responses. *Oxford Journal of Health Policy and Planning*. 2015;30(7):917-927. doi:10.1093/heapol/czu092

29. Petersen I, Lund C, Bhana A, Fisher JA, Consortium MHaPRP. A task shifting approach to primary mental health care for adults in South Africa: human resource requirements and costs for rural settings. *Health Policy Planning*. 2012;27(1):42-51. doi:10.1093/heapol/czr012

30. Petersen I, Hanass-Hancock J, Bhana A, Govender K. Closing the treatment gap for depression co-morbid with HIV in South Africa: Voices of afflicted women. *Health*. 2013;5:557-566. doi:10.4236/health.2013.53A074

31. Blakemore SJ, Mills KL. Is adolescence a sensitive period for sociocultural processing? *Annu Rev Psychol*. 2014;65:187-207.

32. Pantelic M, Boyes M, Cluver L, Meinck F. HIV, violence, blame and shame: pathways of risk to internalized HIV stigma among South African adolescents living with HIV. *J INt AIDS Soc*. 2017;20(21771)

33. Cluver LD, Gardner F, Operario D. Effects of stigma on the mental health of adolescents orphaned by AIDS. *J Adolesc Health*. 2008;42:410-417.

34. Toska E, Cluver LD, Hodes R, Kidia KK. Sex and secrecy: How HIV-status disclosure affects safe sex among HIV-positive adolescents. *AIDS Care*. 2015;27:47-58.

35. Hazra R, Siberry GK, Mofenson LM. Growing up with HIV: children, adolescents, and young adults with perinatally acquired HIV infection. *Annu Rev Med*. 2010;61:169-185.

36. Casale M. The importance of family and community support for the health of HIV-affected populations in Southern Africa: what do we know and where to from here? *British Journal of Health Psychology*. 2015;20(1):21-35. doi:10.1111/bjhp.12127.

37. Casale M. Direct and Indirect Effects of Caregiver Social Support on Adolescent Psychological Outcomes in Two South African AIDS-Affected Communities. *Am J Community Psychol*. 2015;55:336-346.

38. Denison JA. ‘The sky is the limit’: adhering to antiretroviral therapy and HIV self-management from the perspectives of adolescents living with HIV and their adult caregivers. *J Int AIDS Soc*. 2015;18(19358)

39. Organization WH. Making Health Services Adolescent Friendly: Developing National Quality Standards for Adolescent Friendly Health Services. 2012;

40. Hawke LD. What makes mental health and substance use services youth friendly? A scoping review of literature. *BMC Health Services Research*. 2019;19(257)

41. Venturo-Conerly KE. Designing Culturally and Contextually Sensitive Protocols for Suicide Risk in Global Mental Health: Lessons From Research With Adolescents in Kenya. *J Am Acad Child Adolesc Psychiatry*. 2022;61:1074-1077.

42. Banda GT, Banda N, Chadza A, Mthunzi C. Suicide epidemic in Malawi: what can we do? *The Pan African Medical Journal*. 2021;38

43. Centre SAL. Man arrested and convicted for attempting to commit suicide. 2023. <https://www.southernafricalitigationcentre.org/2022/11/10/malawi-man-arrested-and-convicted-for-attempting-to-commit-suicide/>

44. Chibanda D, Weiss HA, Verhey R, et al. Effect of a primary care–based psychological intervention on symptoms of common mental disorders in Zimbabwe: a randomized clinical trial. *Jama*. 2016;316(24):2618-2626.

45. Broström S, Johansson B, Verhey R, Landgren K. “Seeing a Brighter Future”–Experiences of adolescents with common mental disorders receiving the problem-solving therapy “Youth Friendship Bench” in Zimbabwe. *Issues in Mental Health Nursing*. 2021;42(11):1019-1029.

46. Wallén A, Eberhard S, Landgren K. The experiences of counsellors offering problem-solving therapy for common mental health issues at the Youth Friendship Bench in Zimbabwe. *Issues in Mental Health Nursing*. 2021;42(9):808-817.

47. Gaynes BN, Akiba CF, Hosseinipour MC, et al. The Sub-Saharan Africa Regional Partnership (SHARP) for Mental Health Capacity-Building Scale-Up Trial: Study Design and Protocol. *Psychiatric services (Washington, DC)*. Jul 1 2021;72(7):812-821. doi:10.1176/appi.ps.202000003

48. Stockton M, Udedi M, Kulisewa K, et al. The impact of an integrated depression and HIV treatment program on mental health and HIV care outcomes among people newly initiating antiretroviral therapy in Malawi. *PLOS One*. 2020;15(5):e0231872.

49. Bengtson AM, Filipowicz TR, Mphonda S, et al. An Intervention to Improve Mental Health and HIV Care Engagement Among Perinatal Women in Malawi: A Pilot Randomized Controlled Trial. *AIDS and Behavior*. 2023:1-12.

50. Johnson JG, Harris ES, Spitzer RL, Williams JB. The patient health questionnaire for adolescents: validation of an instrument for the assessment of mental disorders among adolescent primary care patients. *Journal of Adolescent Health*. 2002;30(3):196-204.

51. Bell AC, D'Zurilla TJ. Problem-solving therapy for depression: a meta-analysis. *Clinical psychology review*. 2009;29(4):348-353.

52. Nezu AM, Perri MG. Social problem-solving therapy for unipolar depression: an initial dismantling investigation. *Journal of Consulting and Clinical Psychology*. 1989;57(3):408.

53. Dao TT. Friendship Bench Intervention to Address Depression and Improve HIV Care Engagement Among Adolescents Living with HIV in Malawi: Study Protocol for a Pilot Randomized Controlled Trial. *medRxiv*. 2024;doi:doi:10.1101/2024.04.11.24305686

54. Chehil S, Kutcher SP. *Suicide risk management: A manual for health professionals*. John Wiley & Sons; 2012.

55. Sun Life Financial Chair in Adolescent Mental Health. *The Tool for Assessment of Suicide Risk for Adolescents (TASR-A): How to use the TASR - A*. 2007.

56. Horowitz LM. Ask Suicide-Screening Questions (ASQ): a brief instrument for the pediatric emergency department. *Arch Pediatr Adolesc Med*. 2012;166:1170-1176.

57. Larsen DL, Attkisson CC, Hargreaves WA, Nguyen TD. Assessment of client/patient satisfaction: development of a general scale. *Evaluation and program planning*. 1979;2(3):197-207.

58. Kalichman C, Seth, Simbayi L, Cloete A, Mthembu P, Phumelele, Mkhonta N, Ruth, Ginindza G, Themba. Measuring AIDS stigmas in people living with HIV/AIDS: the internalized AIDS-related stigma scale. *AIDS Care*. February 2009 2009;21(1):87-93. doi:10.1080/09540120802032627

59. Rimkeviciene J, O'Gorman J, Hawgood J, De Leo D. Development and validity of the Personal Suicide Stigma Questionnaire (PSSQ): A new tool to assess stigmatization among those who are suicidal. *Crisis: The Journal of Crisis Intervention and Suicide Prevention*. 2019;40(5):317-325. doi:<https://doi.org/10.1027/0227-5910/a000567>

60. Stanley B, Green KL, Ghahramanlou-Holloway, Brenner LA, Brown GK. The construct and measurement of suicide-related coping. *Psychiatry Research*. 2017;258:189-193.

61. Van Orden KA, Witte TK, Gordon KH, Bender TW, Joiner TE. Suicidal desire and the capability for suicide: tests of the interpersonal-psychological theory of suicidal behavior among adults. *J Consult Clin Psychol*. 2008;76:72-83.

62. Rudd DM. Rethinking Hopelessness and Suicide. 2004;

63. Cavazos-Rehg P. The impact of discomfort with HIV status and hopelessness on depressive symptoms among adolescents living with HIV in Uganda. *AIDS Care*. 2021;33:867-872.

64. Dozois DJA, Covin R. The Beck Depression Inventory-II (BDI-II), Beck Hopelessness Scale (BHS), and Beck Scale for Suicide Ideation (BSS). *Comprehensive Handbook of Psychological Assessment*. John Wiley & Sons, Inc.; 2004:50-69.

65. Sancho-Domingo C, Cabrallo JL, Coloma-Carmona A, Buyssee DJ. Brief version of the Pittsburgh Sleep Quality Index (B-PSQI) and measurement invariance across gender and age in a population-based sample. *Psychol Assess*. 2021;3:111-121.

66. Prins A, Bovin MJ, Kimerling R, et al. Primary Care PTSD Screen for DSM-5 (PC-PTSD-5). 2015;

67. Smith P, Schmidt S, Allensworth-Davies D, Saitz R. Primary care validation of a single-question alcohol screening test. *Journal of General Internal Medicine*. 2009;24:783-788.

68. Matson TE. Validity of the single-item screen–cannabis (SIS-C) for cannabis use disorder screening in routine care. *JAMA Network Open*. 2022;5(11):e2239772-e2239772.

69. Wilcox S. S. Social relationships and PTSD symptomatology in combat veterans. *Psychological Trauma: Theory, Research, Practice, and Policy*. 2010;2:175-182.

70. Stewart RC, Umar E, Tomenson B, Creed F. Validation of the multi-dimensional scale of perceived social support (MSPSS) and the relationship between social support, intimate partner violence and antenatal depression in Malawi. *BMC Psychiatry*. 2014;14(180)

71. Gottert A. The People Living with HIV (PLHIV) Resilience Scale: Development and Validation in Three Countries in the Context of the PLHIV Stigma Index. *AIDS Behav*. 2019;23:172-182.

72. Spitzer RL, Kroenke K, Williams JBW, Löwe B. A brief measure for assessing generalized anxiety disorder: the GAD-7. *Arch Intern Med*. 2006;166:1092-1097.

73. Chorpita BF, Yim L, Moffitt C, Umemoto LA, Francis SE. Assessment of symptoms of DSM-IV anxiety and depression in children: a revised child anxiety and depression scale. *Behav Res Ther*. 2000;38:835-855.

74. Association AP. DSM-5 TR Self-Rated Level 1 Cross-Cutting Symptoms Measure--Child Age 11-17. 2013.

75. Clarke DE, Kuhl EA. DSM-5 cross-cutting symptom measures: a step towards the future of psychiatric care? *World Psychiatry*. 2014;13:314-316.

76. Miles MB, Huberman AM. *Qualitative Data Analysis: An Expanded Sourcebook*. SAGE; 1994.

77. Ulin PR, Robinson ET, Tolley EE. *Qualitative Methods in Public Health: A Field Guide for Applied Research.* Joey-Bass; 2005.
